# Factors associated with clinical leadership among cardiovascular nurses in China: a multicentre cross-sectional study

**DOI:** 10.1080/07853890.2026.2678681

**Published:** 2026-06-09

**Authors:** Ligang Wang, Haiyan Yu, Linjing Wu, Xianfeng Wu, Yuan Chen

**Affiliations:** Department of Nursing, Xiamen Cardiovascular Hospital, Xiamen University, Xiamen City, Fujian 361000, China

**Keywords:** Cardiovascular, nurse, clinical leadership, influencing factor, cross-sectional study

## Abstract

**Objectives:**

This study aimed to investigate the current level of clinical leadership among cardiovascular nurses and identify its associated factors.

**Methods:**

A multicentre cross-sectional study was conducted among 411 cardiovascular nurses from three tertiary hospitals in China between January and June 2025. Data were collected using the Clinical Leadership Scale, Nursing Work Environment Scale and General Self-Efficacy Scale. Descriptive statistics, univariate analysis, Pearson correlation and multiple linear regression were used for data analysis.

**Results:**

The mean total score of clinical leadership was 63.53 ± 9.81 (mean item score 4.24 ± 0.82), indicating an above-average level. Higher scores were found in motivating others and role modelling, whereas challenging the status quo was the lowest-performing dimension. Professional title, working department, clinical leadership training, nursing work environment and general self-efficacy were independent positive influencing factors (all *p* < 0.05). Nursing work environment and general self-efficacy were strongly and positively correlated with clinical leadership (*r* = 0.738, 0.584; both *p* < 0.01).

**Conclusions:**

Clinical leadership among cardiovascular nurses is relatively high and is affected by both organisational and individual psychological factors. Targeted leadership training, improved nursing work environment and enhanced self-efficacy can effectively strengthen clinical leadership in cardiovascular nursing practice.

## 1. Introduction

With global social and economic development, the ageing population and accelerating urbanisation, unhealthy lifestyles are becoming increasingly prevalent, and the impact of cardiovascular diseases (CVD) on public health is growing more significant [1]. CVD caused approximately 19.8 million deaths globally in 2022 and generated 396 million disability-adjusted life-years lost. CVD deaths are the leading cause of death among urban and rural residents [2]. In China, the burden of CVD continues to rise, with hypertension, coronary heart disease and stroke imposing substantial pressure on the healthcare system and seriously affecting population health [3]. Given the high morbidity, mortality and complexity of CVD management, the delivery of high-quality nursing care is critically important.

Cardiovascular nurses work in highly specialised, high-acuity, and time-critical clinical environments characterised by rapid patient deterioration, complex hemodynamic instability and the need for immediate decision-making, which place unique demands on nurses and make strong clinical leadership particularly critical for coordinating multidisciplinary care, prioritising interventions and ensuring patient safety compared with less acute or more protocol-driven specialties, alongside responsibilities such as medication management and long-term patient education, which in this context includes providing guidance on medication adherence, lifestyle modification (e.g. diet, physical activity and smoking cessation) and self-management of chronic cardiovascular conditions after discharge – roles that may vary across healthcare systems but are particularly central to cardiovascular nursing practice in China [4].

Clinical leadership is an ambiguous and context-dependent concept. A growing body of literature has recently attempted to clarify this relatively new concept; however, its meaning is still unclear [5]. especially in the hospital context, nurses’ clinical leadership refers to nurses who are directly involved in providing nursing care at the bedside and who exert influence on health care team colleagues to achieve positive patient outcomes, even though no formal authority has been vested in them [6]. which is regarded as one of the important core competencies of clinical nurses and plays an important role in improving the overall quality of nursing and promoting nursing team collaboration [7]. The leadership not only affects the professional level of nurses but also directly relates to the quality of care and nursing management effectiveness of patients [8]. Nurses’ clinical leadership not only influences nurses’ professional development but is also closely associated with care quality and organisational effectiveness [9].

International organisations have emphasised the importance of strengthening nursing leadership. The International Council of Nurses (ICN) developed the Leadership for Change programme to enhance nurses’ leadership capacity and promote healthcare improvement [10], and the World Health Organization (WHO) identified nursing leadership as a key strategic priority in its Global Strategic Directions for Nursing and Midwifery 2021–2025. Although clinical leadership is recognised as a core competency across nursing specialties, its manifestation and influencing factors are highly context-dependent [11]. In cardiovascular settings, nurses face greater clinical uncertainty, rapid decision-making demands and complex interprofessional collaboration compared with many other specialties, which may shape both the development and expression of clinical leadership behaviours. However, existing research has predominantly focused on general nursing populations or specific groups such as ICU nurses and nurse managers, with limited attention to cardiovascular nurses as a distinct professional group. This gap restricts the development of targeted strategies tailored to the unique demands of cardiovascular nursing practice [12]. Therefore, examining clinical leadership specifically in cardiovascular settings is necessary to generate context-specific evidence.

Previous research has shown that factors such as professional title, training experience, nursing work environment and individual psychological characteristics may influence nurses’ clinical leadership levels. A supportive nursing work environment has been associated with improved job satisfaction and professional engagement, which may facilitate leadership behaviours. Additionally, general self-efficacy – defined as an individual’s belief in their ability to organise and execute actions required to manage prospective situations [13]. Higher self-efficacy has been associated with increased confidence, resilience and proactive behaviour in clinical settings. Nurses with stronger self-efficacy are more likely to engage in decision-making, communicate effectively within teams and take initiative in complex situations, all of which are essential components of clinical leadership [14]. Empirical studies have also demonstrated a positive relationship between self-efficacy and leadership behaviours in healthcare professionals. However, evidence regarding this relationship among cardiovascular nurses remains limited, particularly in the context of specialised cardiovascular hospitals.

Despite the increasing burden of CVD and the complex working conditions faced by cardiovascular nurses, there remains a lack of empirical evidence regarding the current level of clinical leadership in this population and the factors influencing it in China. Addressing this gap is essential for developing targeted interventions that strengthen clinical leadership in cardiovascular care settings. Therefore, this study aimed to explore the current status of clinical leadership among cardiovascular nurses and to analyse its influencing factors, including demographic characteristics, nursing work environment and general self-efficacy. The findings may provide a theoretical basis for nursing managers to develop evidence-based strategies to enhance clinical leadership and improve the quality of cardiovascular nursing care.

## 2. Materials and methods

### 2.1. Study design and setting

A multicentre cross-sectional study design was adopted, and the results were reported following the Strengthening the Reporting of Observational Studies in Epidemiology (STROBE) statement [15]. The study was conducted between January 2025 and June 2025 in three large public tertiary A hospitals in Xiamen, Fujian Province, China. These hospitals are major cardiovascular care centres with bed capacities ranging from 600 to 1500, each containing specialised cardiovascular units, including cardiology wards, cardiac surgery wards, cardiovascular intensive care units (ICUs), cardiac catheterisation laboratories and emergency departments. A cluster sampling method was used to recruit cardiovascular nurses from these hospitals. All cardiovascular nurses working in the participating units during the study period were invited to take part. A preprint version of this study has been deposited on Research Square prior to submission [16].

### 2.2. Study participants and inclusion criteria

The inclusion criteria for nurses were as follows: (1) holding a valid nursing qualification certificate; (2) having worked in the cardiovascular field for at least one year; and (3) voluntarily participating in the study. Exclusion criteria included: nurses on leave or external training during the data collection period; nurses from other hospitals undertaking further study or practical training at the participating hospitals; and nurses undergoing probationary training.

### 2.3. Sample size estimation

Based on the sample size estimation method for Kendall correlations [17], the sample size was estimated to be at least five to ten times the number of observed variables. To account for potential sample loss and invalid cases, a 10% attrition rate was added. Given that the study included 60 variables, the required sample size for the calculation is 310 cases. The final sample size of 411 exceeded the minimum requirement.

### 2.4. Measures

Four instruments were used in this study. Permission for use was obtained from the original authors or authorised translators where required.

#### 2.4.1. Demographics and work characteristics

A structured questionnaire developed by the researchers collected demographic and professional information, including gender, age, educational level, professional title, department and whether the nurse had received clinical leadership training. In China, the nursing professional title system includes three primary levels: Nurse (entry-level registered nurse); Senior nurse (mid-level nurse with more clinical experience and responsibility); Supervisory nurse and above (advanced-level nurse with extended clinical expertise and leadership responsibilities). These titles are based on years of experience, professional examinations and institutional evaluation standards. In the present study, age was categorised into three groups: 20–29, 30–40 and ≥ 41 years, based on common career stage divisions for clinical nurses in China, where the 30–40 age period typically represents a transition from proficiency to advanced expertise and potential leadership development [18]. Total work experience was classified as 1–5, 6–10, 11–15 and ≥16 years, with the thresholds of 5, 10 and 15 years representing typical milestones for professional skill maturation and career advancement in Chinese nursing practice [19].

#### 2.4.2. Clinical leadership scale

The Clinical Leadership Scale was originally developed by Canadian scholar Patrick [20] and later translated and validated in Chinese [21]. The scale consists of 15 items across five dimensions: challenging the current situation (three items), inspiring the vision (three items), setting an example (three items), inspiring others (three items) and motivating people (three items). Each item is scored on a five-point Likert scale ranging from 1 (‘almost never’) to 5 (‘almost always’), with higher total scores indicating stronger clinical leadership. The Chinese version of the scale demonstrated good internal consistency (Cronbach’s α = 0.942) and satisfactory construct validity (CFI = 0.941, RMSEA = 0.079) in the validation study. In the present study, the Cronbach’s *ɑ* coefficient of the scale was 0.945 [22], indicating excellent reliability.

#### 2.4.3. Nursing work environment scale

The Nursing Work Environment Scale was developed by Jing Shao in 2016 [23]. It includes 30 items across seven dimensions: career development (six items), leadership and management (six items), medical relationship (four items), recognition atmosphere (four items), professional autonomy (four items), basic guarantee (three items) and sufficient manpower (three items). The questionnaire was scored on a positive six-point Likert scale, the total score ranges from 30 to 180 points, with higher scores indicating better nursing work environment. In the original development study, the scale showed high internal consistency (Cronbach’s α = 0.946) and good model fit (CFI = 0.930, RMSEA = 0.064) [24].

#### 2.4.4. General self-efficacy scale

The general self-efficacy scale was originally developed by Kaikang Wang et al. [25] with a total of ten items, to measure the self-efficacy level of participants. All options of this questionnaire were scored on a positive four-point Likert scale, with scores ranging from 1 to 4 for ‘completely incorrect’ to ‘completely correct’. The higher the score, the better the individual’s self-efficacy level. In the original development study, the scale showed high internal consistency (Cronbach’s α = 0.965) and good test-retest reliability (*r* = 0.83) [26].

### 2.5. Data collection procedures

A pilot test was first conducted among 30 cardiovascular nurses to ensure the clarity and comprehensibility of all questionnaire items; all participants indicated that the items were understandable. No modifications were made to the instruments based on the pilot test. For the main survey, a cluster sampling method was adopted. Clusters were defined as each cardiovascular-related unit (e.g. cardiology ward, cardiac surgery ward, cardiovascular intensive care unit, cardiac catheterisation laboratory and emergency department) within the three participating tertiary A hospitals. All cardiovascular nurses who met the inclusion criteria and were working in the selected clusters during the study period were invited to participate. This approach ensured that the sample represented the entire target population of cardiovascular nurses across different unit types. To ensure that only eligible nurses participated in the survey, a two-step verification process was implemented. First, before distributing the questionnaire link, the head nurse of each participating unit reviewed the list of unit members and confirmed that all potential respondents held a valid nursing qualification certificate and had at least one year of experience in the cardiovascular field. Second, the online questionnaire (created using the Questionnaire Star platform, Ranxing, Changsha, China) included a mandatory screening page at the beginning, which explicitly stated the inclusion criteria and asked participants to self-confirm their eligibility before proceeding. Only those who confirmed eligibility could access the subsequent items. The questionnaire link was distributed *via* WeChat (Tencent, Shenzhen, China) by the head nurses to the department’s WeChat group. A standardised instruction page was provided at the beginning of the questionnaire, explaining the study purpose, filling instructions and confidentiality assurances. All nurses completed the questionnaire anonymously and independently. To prevent duplicate responses, the questionnaire could only be submitted once from the same account and device. All questions were set as mandatory fields to ensure complete data collection. A total of 430 cardiovascular nurses were invited to participate in the study, of whom 423 returned questionnaires (response rate: 98.37%). After data screening, 12 questionnaires were excluded due to invalid response patterns (e.g. identical answers across all items), resulting in 411 valid questionnaires and a valid response rate of 95.58%.

### 2.6. Ethical considerations

This study was approved by the Ethics Committee of Xiamen Cardiovascular Hospital, Xiamen University, China No. (2024) Medical Ethics Committee No. (5). And the study was conducted in accordance with the Declaration of Helsinki. Participation was voluntary, and informed consent was obtained electronically from all participants before data collection. Confidentiality and anonymity were strictly maintained, and no identifiable personal information was collected. All personal information was strictly protected, and the data were used solely for research analysis

### 2.7. Statistical analysis

SPSS software (V.26.0) was used for data interpretation and statistical analysis. Counting data such as title and gender were described by frequency and component ratio. After the normality test, the data were normally distributed, so the measurement data, such as age and working years, were described by means and standard deviations (*SD*), and the correlation between variables was tested by *Pearson correlation analysis.* Comparisons between groups were done using analysis of variance (ANOVA), and independent samples t-test with pairwise comparisons was conducted using the SNK-*q* test. Variables with *p* < 0.05 in univariate analysis were entered into a multiple linear regression model to identify independent influencing factors. Statistical significance was set at *p* < 0.05.

## 3. Results

### 3.1. Demographic characteristics

Of the participants, 411 young cardiovascular nurses were finally included, of whom 346 (84.18%) were female, and 65 (15.82%) were male. The majority of participants were aged 30–40 years (47.45%), and the largest proportion held the title of senior nurse (47.45%). There were statistically significant differences in the clinical leadership scale scores of nurses working in the cardiovascular field based on their gender, professional title, department, and whether they had undergone training on clinical leadership (*p* < 0.05) (Table 1).

**Table 1.** Single factor analysis of general information and clinical leadership scores of cardiovascular nurses(*n* = 411, $\bar{x}\pm s$ ).

Items	Number of cases(%)	score	F/t value	*P* value
**Gender**				
Male	65(15.82)	61.14 ± 10.93	*t* = −2.147^#^	0.032
Female	346(84.18)	63.97 ± 9.54		
**Age group(years)**				
20∼29	156(37.96)	62.91 ± 9.46	*F* = 1.769*	0.172
30∼40	195(47.45)	63.36 ± 10.16		
≥41	60(14.6)	65.67 ± 9.44		
**Title**				
Nurse	33(8.03)	64.45 ± 10.63	*F* = 4.226*	0.015
Senior nurse	195(47.45)	62.06 ± 9.21		
Supervisory Nurse and above	183(44.53)	64.92 ± 10.12		
**Duration of overall work experience(years)**				
1∼5	108(26.28)	63.29 ± 9.87	*F* = 2.367*	0.07
6∼10	157(38.2)	62.2 ± 10.20		
11∼15	59(14.36)	65.56 ± 8.81		
≥16	87(21.27)	64.84 ± 9.45		
**Department**				
Internal Medicine	164(39.9)	64.17 ± 9.77	*F* = 2.362*	0.03
Surgery	50(12.17)	63.06 ± 9.07		
Pediatrics	4(0.97)	62.00 ± 3.37		
Intensive Care Unit	76(18.49)	60.99 ± 9.76		
Operating room	33(8.03)	65.3 ± 9.90		
Emergency Department	21(5.11)	59.52 ± 7.37		
Other	63(15.33)	65.78 ± 10.74		
**Whether underwent clinical teaching instructor**				
Yes	205(49.88)	65.82 ± 9.96	*t* = 2.615^#^	0.039
No	206(50.12)	63.23 ± 9.68		
**Whether underwent training on clinical leadership**				
Yes	65(15.82)	65.29 ± 8.87	*t* = 1.585^#^	0.014
No	346(84.18)	63.19 ± 9.96		
**Educational qualification**				
Associate degree	33(8.03)	65.73 ± 11.19	*F* = 1.08*	0.34
Bachelor’s degree	357(86.86)	63.41 ± 9.76		
Master’s degree and above	21(5.11)	62.10 ± 8.23		

Note: t: two independent samples t-test, F: one-way ANOVA.

### 3.2. Correlation analysis of clinical leadership, nursing work environment and self-efficacy among cardiovascular nurses

The results showed that the total scores of the clinical leadership, nursing work environment and self-efficacy were 63.53 ± 9.81, 156.58 ± 23.47 and 32.60 ± 5.59, respectively, the score of each scale is shown in Table 2. Pearson correlation analysis showed that work environment and self-efficacy were positively correlated with clinical leadership scores, as shown in Table 3.

**Table 2.** The clinical leadership scale, the nursing work environment scale and the general self-efficacy scale scores among cardiovascular nurses.

Items	Number of entries	Score	Equal distribution of items
Total score of the Clinical Leadership Scale	15	63.53 ± 9.81	4.24 ± 0.82
Challenge the current situation	3	11.83 ± 2.47	3.94 ± 0.91
Motivating vision	3	12.25 ± 2.24	4.08 ± 0.84
Set an example	3	13.13 ± 2.20	4.38 ± 0.76
Inspiring others	3	13.09 ± 2.12	4.36 ± 0.74
Motivating people	3	13.23 ± 2.08	4.41 ± 0.74
Total score of the Nursing Work Environment Scale	30	156.58 ± 23.47	5.23 ± 0.86
Career development	6	32.11 ± 4.83	5.35 ± 0.90
Leadership and management	6	32.07 ± 5.22	5.34 ± 0.92
Medical relationship	4	20.64 ± 3.75	5.16 ± 0.98
Recognition atmosphere	4	20.73 ± 3.57	5.18 ± 0.96
Professional autonomy	4	21.04 ± 3.36	5.26 ± 0.89
Basic guarantee	3	15.37 ± 2.84	5.12 ± 1.01
Sufficient manpower	3	14.62 ± 3.23	4.87 ± 1.12
Total score of the General Self-efficacy Scale	10	32.60 ± 5.59	3.26 ± 0.65

**Table 3.** Correlation analysis of clinical leadership, nursing work environment and self-efficacy among cardiovascular nurses.

	Level of clinical leadership	Total score of nursing work environment	Total score of general self-efficacy
Level of clinical leadership	1		
Total score of nursing work environment	0.738**	1	
Total score of general self-efficacy	0.584**	0.644**	1

Note: **p < 0.01.

### 3.3. Multiple linear regression analysis on factors influencing clinical leadership among cardiovascular nurses

Multivariable linear regression analysis was performed with the total score of the clinical leadership among cardiovascular nurses as the dependent variable, the items with *p* < 0.05 as the independent variable. The analysis showed the gender, title, department and whether underwent training on clinical leadership, the nursing work environment and the general self-efficacy were positive influencing factors of clinical leadership, as shown in Table 4.

**Table 4.** Multivariate linear regression analysis of influencing factors for clinical leadership among cardiovascular nurses (*n* = 411).

Items	B Value	Standard error	β	t value	*P* value	VIF
Constant	17.418	7.396		2.355	0.019	
Gender (reference: female)	2.703	1.695	0.055	1.595	0.06	1.103
Title	1.026	1.193	0.036	0.86	0.047	1.61
Department	−0.154	0.27	−0.02	−0.571	0.036	1.07
Whether underwent clinical teaching instructor Yes (reference: No)	0.23	1.474	0.006	0.156	0.047	1.566
Whether underwent training on clinical leadership Yes (reference: No)	−0.841	1.663	−0.017	−0.506	0.022	1.062
Nursing work environment level	0.455	0.034	0.6	13.409	<0.001	1.822
Self-efficacy level	0.598	0.142	0.188	4.212	<0.001	1.812

Note: *R*2 = 0.558, adjusted *R*2 = 0.550, *F* = 72.642, *p* < 0.001.

VIF: Variance inflation factor.

## 4. Discussion

This multicentre cross-sectional study provides empirical evidence on clinical leadership among cardiovascular nurses in China. The main findings can be summarised as follows: (1) the overall level of clinical leadership was above average, with the highest scores in ‘motivating people’ and ‘setting an example’, and the lowest in ‘challenging the status quo’; (2) a supportive nursing work environment and higher general self-efficacy were strongly and positively correlated with clinical leadership; and (3) professional title, working department, clinical leadership training and serving as a clinical teaching instructor were independent positive influencing factors. These findings highlight that clinical leadership in cardiovascular nursing is shaped by both organisational and individual psychological factors

### 4.1. Overall level and dimensional differences of clinical leadership

Compared with previous studies conducted among ICU nurses [27] and newly qualified nurses [28], the mean item score of 4.24 indicates that cardiovascular nurses frequently perform leadership behaviours in daily practice. However, the score was slightly higher than that reported in general nursing populations, which may reflect the unique demands of cardiovascular settings. High-acuity, rapid clinical deterioration and complex multidisciplinary coordination require cardiovascular nurses to assume informal leadership roles routinely. Interestingly, the highest scores were observed in ‘motivating people’ and ‘setting an example’, whereas ‘challenging the status quo’ scored the lowest. This pattern can be explained by the nature of cardiovascular nursing. Extended treatment courses and complex patient profiles necessitate tight team coordination; nurses consistently provide emotional support to patients and encourage colleagues under high workload, thereby demonstrating motivation and role modelling [29]. Conversely, the low score in challenging the status quo likely stems from strict adherence to standardised protocols (e.g. emergency responses for arrhythmias and volume management in heart failure) [30] to minimise clinical risks [31]. Moreover, traditional hierarchical management structures in Chinese healthcare settings provide few opportunities or incentives for frontline nurses to engage in system improvement or process innovation [32]. This finding echoes Bai et al. [33], who reported limited competence in promoting change among clinical nurses. Targeted training programs that foster innovative thinking and psychological safety are needed to empower cardiovascular nurses to challenge existing practices appropriately.

### 4.2. Nursing work environment as a key organisational driver

Our study found that a better nursing work environment was strongly associated with higher clinical leadership (*r* = 0.738, *p* < 0.01), and it remained the most influential factor in the regression model (*β* = 0.600, *p* < 0.001). This finding aligns with the socio-ecological perspective that leadership behaviours are enabled or constrained by contextual factors. A healthy work environment provides career development opportunities, recognition, professional autonomy and adequate staffing [23], which collectively increase job satisfaction, alleviate burnout and reduce turnover intention among cardiovascular nurses [34]. More importantly, nurses who perceive a supportive environment demonstrate greater work motivation, proactive skill development and effective interprofessional collaboration, all of which are behavioural precursors to clinical leadership [35]. This study confirms that the nursing work environment is not merely a background condition but an active determinant of clinical leadership. Therefore, hospital administrators should prioritise establishing a healthy work environment [36] – including fair recognition systems, sufficient human resources and shared governance structures – to create favourable conditions for leadership emergence among cardiovascular nurses [37].

### 4.3. General self-efficacy as an individual psychological resource

Consistent with social cognitive theory [38] and previous empirical studies [39], higher general self-efficacy was significantly associated with greater clinical leadership (*β* = 0.188, *p* < 0.001). Nurses with strong self-efficacy maintain a positive and optimistic attitude when managing clinical emergencies, demonstrating greater sensitivity to environmental changes and greater confidence in their ability to influence outcomes. In this study, 73.72% of participants had more than five years of clinical experience. As experience accumulates, both professional knowledge and practical skills improve, enabling nurses to accurately assess their competencies and address various issues with confidence, thereby enhancing self-efficacy. These nurses are more likely to participate in clinical decision-making, engage in effective team communication and collectively achieve organisational goals [40]. This finding suggests that self-efficacy is not a fixed trait but a modifiable psychological resource. Nursing administrators should regularly conduct self-efficacy-enhancing interventions, such as mastery experiences [41] (e.g. simulation training), vicarious experiences (e.g. peer role modelling) and constructive feedback, to motivate nurses and enable them to practice at higher levels of expertise [40].

### 4.4. The role of clinical teaching instructor and leadership training

Serving as a clinical teaching instructor emerged as an independent factor associated with higher clinical leadership. This finding is consistent with Xu [21] and can be explained by the rigorous selection and training process for teaching instructors, which enhances their professional skills, analytical abilities and problem-solving efficacy [42]. More importantly, by mentoring new and intern nurses and modelling best practices, these instructors exert leadership through example, influencing colleagues’ professional attitudes and behaviours [43]. We recommend that nursing managers incorporate clinical leadership competency into the selection and assessment criteria for clinical teaching instructors, thereby encouraging all nurses to focus on leadership development [44].

Interestingly, while participation in clinical leadership training was statistically significant, its association in the regression model was negative (*β* = −0.017, *p* = 0.022). This counterintuitive finding may be explained by several factors. First, nurses who have received formal training may have higher expectations of leadership performance and therefore rate themselves more critically (response shift bias). Second, only 15.82% of participants had received such training, a much lower proportion than reported in related studies [45], which may limit the observable positive impact. Third, existing leadership training programs in China predominantly target nursing managers rather than frontline clinical nurses [46], and the content may not be tailored to the specific needs of cardiovascular nurses. This finding should be interpreted cautiously and highlights the urgent need to develop context-specific, accessible leadership training for clinical nurses. Future research using longitudinal designs is needed to evaluate the causal effects of well-designed leadership training on clinical leadership outcomes [47].

### 4.5. Implications for practice and policy

Taken together, our findings indicate that strengthening clinical leadership in cardiovascular nursing requires a multidimensional approach. At the organisational level, hospital administrators should improve the nursing work environment by ensuring adequate staffing, providing recognition and fostering professional autonomy. At the individual level, interventions to enhance self-efficacy – such as mastery experiences, role modelling and constructive feedback – should be integrated into continuing education. Finally, accessible and context-specific clinical leadership training programs should be developed for frontline cardiovascular nurses, moving beyond the traditional focus on nurse managers alone. Such integrated efforts may contribute to more sustainable leadership development and ultimately improve the quality of cardiovascular nursing care.

### 4.6. Study limitations

This study has several limitations that should be considered when interpreting the results. First, participants were recruited from tertiary hospitals within a single city using convenience sampling, which may limit the representativeness of the sample and constrain the generalisability of the findings to other regions, institutional contexts, or healthcare systems. Second, the cross-sectional design precludes the establishment of causal relationships between clinical leadership and its associated factors. Third, all variables were measured self-reported questionnaires, which may be subject to common method variance and social desirability bias. Future research employing multicentre designs, longitudinal approaches and multi-source assessment methods is warranted to enhance the robustness and external validity of these findings.

## 5. Conclusions

Clinical leadership behaviours among cardiovascular nurses were generally performed frequently in daily practice, with stronger performance in motivational and role-modelling dimensions. Both organisational factors (clinical teaching role, leadership training and work environment) and individual factors (self-efficacy) were independently associated with leadership levels. These findings suggest that strengthening clinical leadership in cardiovascular nursing may require attention to both workplace conditions and nurses’ psychological resources. Further multi-centre and longitudinal studies are needed to confirm these associations and explore causal relationships.

## Supplementary Material

Open Science Badges Disclosure Form - Annals of Medicine - IANN-2026-1861.R1.docx

## Data Availability

The dataset generated and/or analysed during the current study is openly available in the Science Data Bank at https://doi.org/10.57760/sciencedb.35497.

## Open scholarship

This article has earned the Center for Open Science badges for Open Data and Open Materials through Open Practices Disclosure. The data and materials are openly accessible at https://doi.org/10.57760/sciencedb.35497 and https://doi.org/10.57760/sciencedb.35497.

## References

[1] Mensah GA, Fuster V, Murray CJL, et al. Global burden of cardiovascular diseases and risks, 1990–2022. J Am Coll Cardiol. 2023;82(25):2350–2473. doi:10.1016/j.jacc.2023.11.007. 38092509 PMC7615984

[2] Vaduganathan M, Mensah GA, Turco JV, et al. The global burden of cardiovascular diseases and risk: a compass for future health. J Am Coll Cardiol. 2022;80:2361–2371. 36368511 10.1016/j.jacc.2022.11.005

[3] Center for Cardiovascular Diseases the Writing Committee of the Report on Cardiovascular Health and Diseases in China N , Report on cardiovascular health and diseases in China 2023: an updated summary. Biomed Environ Sci. 2024;37:949–992. 39401992 10.3967/bes2024.162

[4] Zaghini F, Fiorini J, Moons P, et al. Cardiovascular nurses and organizational well-being: a systematic review. Eur J Cardiovasc Nurs. 2024;23(3):213–220. doi:10.1093/eurjcn/zvad078. 37561990

[5] Larsson IE, Sahlsten MJ. The staff nurse clinical leader at the bedside: Swedish registered nurses’ perceptions. Nurs Res Pract. 2016;2016:1797014. doi:10.1155/2016/1797014. 28044103 PMC5164887

[6] Chávez EC, Yoder LH. Staff nurse clinical leadership: a concept analysis. Nurs Forum. 2015;50(2):90–100. doi:10.1111/nuf.12100. 24935803

[7] Enghiad P, Venturato L, Ewashen C. Exploring clinical leadership in long-term care: an integrative literature review. J Nurs Manag. 2022;30(1):90–103. doi:10.1111/jonm.13470. 34541738

[8] James AH, Bennett CL, Blanchard D, et al. Nursing and values-based leadership: a literature review. J Nurs Manag. 2021;29(5):916–930. doi:10.1111/jonm.13273. 33484188

[9] Cai Y, Li Q, Cao T, et al. Nurses’ work engagement: the influences of ambidextrous leadership, clinical nurse leadership and workload. J Adv Nurs. 2023;79(3):1152–1161. 34723406 10.1111/jan.15086

[10] Ferguson SL, Al Rifai F, Maay’a M, et al. The ICN leadership for change^™^ programme–20 years of growing influence. Int Nurs Rev. 2016;63(1):15–25. doi:10.1111/inr.12248. 26923323 PMC5515374

[11] Boamah S. Linking nurses’ clinical leadership to patient care quality: the role of transformational leadership and workplace empowerment. Can J Nurs Res. 2018;50(1):9–19. doi:10.1177/0844562117732490. 29108423

[12] Ferreira TDM, de Mesquita GR, de Melo GC, et al. The influence of nursing leadership styles on the outcomes of patients, professionals and institutions: an integrative review. J Nurs Manag. 2022;30(4):936–953. doi:10.1111/jonm.13592. 35293055

[13] Shoji K, Cieslak R, Smoktunowicz E, et al. Associations between Job Burnout and self-efficacy: a meta-analysis. Anxiety Stress Coping. 2016;29(4):367–386. doi:10.1080/10615806.2015.1058369. 26080024

[14] Bonsaksen T. Predictors of general self-efficacy and self-esteem in occupational therapy students: a cross-sectional study. Occup Ther Ment Health.2016; 32(3), 226–242.doi:10.1080/0164212X.2015.1055536.

[15] Cuschieri S. The STROBE guidelines. Saudi J Anaesth. 2019;13(Suppl 1):S31–S34. doi:10.4103/sja.SJA_543_18. 30930717 PMC6398292

[16] Wang LG, Yu HY, Wu LJ, et al. Analysis on the current situation and influencing factors of clinical leadership of cardiovascular nurses in China, a cross-sectional study. Res Sq. 2025. doi:10.21203/rs.3.rs-7589353/v1.

[17] Li Z, Liu Y. Research methods of nursing. Beijing: People’s Medical Publishing House; 2018.

[18] Tlili MA, Haoues M, Kamoun H, et al. Exploring how organizational support shapes nurses’ clinical leadership: evidence from Tunisia. BMC Nurs. 2026;25(1):130. doi:10.1186/s12912-026-04294-8. 41514229 PMC12882198

[19] Gillet N, Fouquereau E, Bonnaud-Antignac A, et al. The mediating role of organizational justice in the relationship between transformational leadership and nurses’ quality of work life: a cross-sectional questionnaire survey. Int J Nurs Stud. 2013;50(10):1359–1367. doi:10.1016/j.ijnurstu.2012.12.012. 23298792

[20] Patrick. The effect of nursing leadership and structural empowerment on staff nurse clinical leadership. Canada: The University of Western Ontario; 2010.

[21] Xu K. Analysis of the Current State of Clinical Leadership Among Nurses in Tertiary Hospitals in China. Beijing: Peking Union Medical College; 2021.

[22] Carlson MA, Morris S, Day F, et al. Psychometric properties of leadership scales for health professionals: a systematic review. Implement Sci. 2021;16(1):85. doi:10.1186/s13012-021-01141-z. 34454567 PMC8403357

[23] Shao Jing Y, Zhihong, T, Leiwen. Development and reliability and validity study of the nursing work environment scale. Chin J Nurs. 2016;51:142–147.

[24] Shao J, Tang L, Ye Z. Measuring the nursing work environment in mainland China: further development of the Chinese Nursing Work Environment Scale. Nurs Res. 2017;66(4):311–322. doi:10.1097/NNR.0000000000000221. 28410271

[25] Caikang W, Zhongfeng H, Yong L. A study on the reliability and validity of the general self-efficacy scale. Appl Psychol; 2001;37–40.

[26] Galiana L, Sánchez-Ruiz J, Gómez-Salgado J, et al. Validation of the Spanish version of the five-item general self-efficacy (GSE) scale in a sample of nursing students: evidence of validity, reliability, longitudinal invariance and changes in general self-efficacy and resilience in a two-wave cross-lagged panel model. Nurse Educ Pract. 2024;74:103865. doi:10.1016/j.nepr.2023.103865. 38128375

[27] Zhao Y, Qu H, Chu J. Analysis of the current status and influencing factors of clinical leadership among ICU Nurses in 18 grade a tertiary general hospitals in Shandong province. Chin Nurs Manag. 2022;22:908–913.

[28] Sa X, Ruijie T, Huiping W. Analysis of the current status and influencing factors of clinical leadership among newly qualified nurses with master’s degrees. J Nurse Educ. 2025;40:123–128.

[29] Morrison L, Johnston B, Cooper M. Mixed methods systematic review: factors influencing research activity among nurses in clinical practice. J Clin Nurs. 2022;31(17–18):2450–2464. doi:10.1111/jocn.16133. 34820932

[30] Zaghini F, Biagioli V, Fiorini J, et al. Work-related stress, job satisfaction, and quality of work life among cardiovascular nurses in Italy: structural equation modeling. Appl Nurs Res. 2023;72:151703. doi:10.1016/j.apnr.2023.151703. 37423684

[31] Liao H, Lou L. Impact of safety risk defense mechanism in operating room nursing on quality and risk incidents. Risk Manag Healthc Policy. 2025;18:1827–1835. doi:10.2147/RMHP.S508662. 40496193 PMC12149271

[32] Mariano MEM, Woodman A, Al Zahrani EM, et al. Turnover-attachment motive of Saudi Arabia nursing workforce: a cross-sectional study. Nurs Open. 2023;10(2):988–997. doi:10.1002/nop2.1366. 36109849 PMC9834159

[33] Bai Q, Bai Y. Heterogeneity of differential atmosphere perception and its relationship with organizational silence among Chinese nurses: a cross-sectional study using latent profile analysis. Front Psychol. 2025;16:1592094. doi:10.3389/fpsyg.2025.1592094. 40497100 PMC12149132

[34] Correia LL, Pereira JDS, Gasparino RC. Influence of the work environment on patient safety and stress among healthcare professionals. Rev Esc Enferm USP. 2025;59:e20240420. doi:10.1590/1980-220X-REEUSP-2024-0420en. 40590535 PMC12211961

[35] Zhang F, Huang L, Fei Y, et al. Impact of caring leadership on nurses’ work engagement: examining the chain mediating effect of calling and affective organization commitment. BMC Nurs. 2024;23(1):716. doi:10.1186/s12912-024-02388-9. 39370507 PMC11456229

[36] Hayman LL, Berra K, Fletcher BJ, et al. The role of nurses in promoting cardiovascular health worldwide: the global cardiovascular nursing leadership forum. J Am Coll Cardiol. 2015;66(7):864–866. doi:10.1016/j.jacc.2015.06.1319. 26271070

[37] Halm MA. The impact of the work environment on patients, families, and care delivery in critical care. Crit Care Nurs Clin North Am. 2025;37(4):543–565. doi:10.1016/j.cnc.2025.07.001. 41193149

[38] Shirey MR. Self-efficacy and the nurse leader. Nurse Lead. 2020;18(1),48-53. doi:10.1016/j.mnl.2020.05.001.

[39] Alilyyani B, Althobaiti E, Al-Talhi M, et al. Nursing experience and leadership skills among staff nurses and intern nursing students in Saudi Arabia: a mixed methods study. BMC Nurs. 2024;23(1):87. doi:10.1186/s12912-024-01750-1. 38308273 PMC10835976

[40] Tomita R. The relationship between general self-efficacy and nursing practice competence for second-year nurses: empirical quantitative research. Nurs Open. 2024;11(7):e2233. doi:10.1002/nop2.2233. 38961662 PMC11222662

[41] Rodríguez-García MC, Márquez-Hernández VV, Belmonte-García T, et al. Original research: how magnet hospital status affects nurses, patients, and organizations: a systematic review. Am J Nurs. 2020;120(7):28–38. doi:10.1097/01.NAJ.0000681648.48249.16. 32541337

[42] Lien RY, Cheng CG, Hung SH, et al. The effect of the knowledge, skills, and attitudes from nurse training using in situ simulation in an intensive care unit. Healthcare (Basel). 2023;11(21). doi:10.3390/healthcare11212851. PMC10649282 37957996

[43] Hossny EK, Sabra HE. The attitudes of healthcare professionals towards nurse-physician collaboration. Nurs Open. 2021;8(3):1406–1416. doi:10.1002/nop2.756. 33378598 PMC8046133

[44] Choi H, Jeon Y, Lee U, et al. Technology-based interactive communication simulation for Korean nurses: A randomized controlled repeated-measures design. Nurse Educ Today. 2023;128:105879. doi:10.1016/j.nedt.2023.105879. 37352764

[45] Shuang S, Zhenhui T, Jialei F. Analysis of current practices and influencing factors of leadership behaviour among head nurses. Chin Nurs Manag. 2024;24:1625–1629.

[46] Bagga R, Jaiswal V, Tiwari R. Role of directorates in promoting nursing and midwifery across the various States of India: call for leadership for reforms. Indian J Community Med. 2015;40(2):90–96. doi:10.4103/0970-0218.153870. 25861169 PMC4389509

[47] Dirik HF, Seren Intepeler S. An authentic leadership training programme to increase nurse empowerment and patient safety: A quasi-experimental study. J Adv Nurs. 2024;80(4):1417–1428. 37921089 10.1111/jan.15926

