# Supplementary material for: Factors associated with clinical leadership among cardiovascular nurses in China: a multicentre cross-sectional study
Source: Ann Med. 2026 Jun 9;58(1):2678681. doi: 10.1080/07853890.2026.2678681 (PMC13250876; doi:10.1080/07853890.2026.2678681)
Supplement: Open Science Badges Disclosure Form - Annals of Medicine - IANN-2026-1861.R1.docx [file IANN_A_2678681_SM3564.docx]

**Open Science Badges at Taylor & Francis
Author Disclosure Form**

| **NOTICE TO EDITORIAL OFFICE**  PLEASE COMPLETE **SECTION B** OF THIS FORM **BEFORE** EXPORTING WITH FINAL FILES |
| --- |

***SECTION A: Disclosure Statement (to be completed by authors)***

**Manuscript title:** **Factors Associated with Clinical Leadership among Cardiovascular Nurses in China: A Multicentre Cross-Sectional Study**

**Corresponding author:** Yuan Chen

Articles accepted to Annals of Medicine are eligible to earn badges that recognize open scientific practices: Open Data, Open Materials, Preregistered or Preregistered+. Please read more about the badges on our [Author Services](https://authorservices.taylorandfrancis.com/open-science-badges/) site. You can also find information on the [Center for Open Science](https://osf.io/tvyxz/) website.

|  | Please check this box if you are interested in participating. |
| --- | --- |

To apply, please read the [qualifying criteria for each badge](https://osf.io/tvyxz/wiki/1.%20View%20the%20Badges/), then complete the information below and return this form to the Journal Editor of Annals of Medicine ( IANN-peerreview@journals.tandf.co.uk ), or upload the file to the Peer Review System with your revised manuscript (if applicable).

Please note: To qualify for a badge, you must provide a URL, DOI, or other permanent path for accessing the specified information in a public, open-access repository. **Qualifying public, open-access repositories are committed to preserving data and/or materials and keeping them publicly accessible via the web in perpetuity.** **Personal websites and most departmental websites do not qualify as repositories.**

Please select desired badge(s) and provide the information as requested:

|  | **Open Materials Badge**  By checking this box, you confirm that the materials provided are sufficient for an independent researcher to reproduce the data collection procedures of the study.  Provide the URL, DOI, or other **permanent path** for accessing the materials in a **public, open-access repository**, such as those suggested by [re3data.org](http://re3data.org): https://doi.org/10.57760/sciencedb.35497 |
| --- | --- |
|  | **Open Data Badge**  By checking this box, you confirm that:   - there is sufficient information for an independent researcher to reproduce **all of the reported results**, including codebook if relevant. - the data made available in a public, open-access repository conform to the ethical guidelines for data handling and archiving as specified in the guidelines of your institution’s Institutional Review Board, Research Ethics Committee, or an equivalent research committee.   Provide the URL, DOI, or other **permanent path** for accessing the materials in a **public, open-access repository**, such as those suggested by [re3data.org](http://re3data.org): **https://doi.org/10.57760/sciencedb.35497** |

|  | **Preregistered Plan Badge:**  Please check the box to apply and provide responses to the disclosure questions below:   1. Provide the URL, DOI, or **other permanent path** to the registration (and, if applicable, the analysis plan) in a **public, open access repository**: 2. Was the plan registered prior to examination of the data or observing the outcomes? If no, explain: 3. Were there additional registrations for the study other than the one reported? If yes, provide links and explain: |
| --- | --- |

Preregistration is **invalidated** if (1) is not provided, or (3) is answered “yes” without strong justification.

|  | **Preregistered+ Analysis Plan Badge:**  Please check the box to apply and provide responses to the disclosure questions below:   1. Provide the URL, DOI, or **other permanent path** to the registration (and, if applicable, the analysis plan) in a **public, open access repository**: 2. Was the plan registered prior to examination of the data or observing the outcomes? If no, explain: 3. Were there additional registrations for the study other than the one reported? If yes, provide links and explain: 4. Were there any changes to the preregistered analysis plan for the primary confirmatory analysis? If yes, explain: 5. Are all of the analyses described in the registered plan reported in the article? If no, explain: |
| --- | --- |

***Authors are accountable to the community for disclosure accuracy.*** *By signing below, authors affirm that the above information is accurate and complete, that any third-party material has been reproduced or otherwise made available only with the permission of the original author or copyright holder, and that publicly posted data do not contain information that would allow individuals to be identified without consent.*

**Name:** **LIgang Wang**

**Date:** **23/4/2026**

***SECTION B: Typesetter Instructions (to be completed by Editorial Office)***

1. *SPlease select which Open Science Badge(s) has/have successfully been achieved:*

| 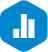 | Open Data | 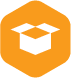 | Open Materials | 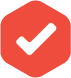 | Preregistered | 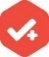 | Preregistered+ |
| --- | --- | --- | --- | --- | --- | --- | --- |

***Note to Typesetters: the corresponding images should appear with the selected text below.***

1. *Please select the appropriate text to accompany the images on the published article, and add the appropriate links providing by the author in SECTION A:*

|  | This article has earned the [Center for Open Science](https://osf.io/tvyxz/wiki/1.%20View%20the%20Badges/) badge for Open Data. The data are openly accessible at https://doi.org/10.57760/sciencedb.35497   . |
| --- | --- |
|  | This article has earned the [Center for Open Science](https://osf.io/tvyxz/wiki/1.%20View%20the%20Badges/) badge for Open Materials. The materials are openly accessible at https://doi.org/10.57760/sciencedb.35497       . |
|  | This article has earned the [Center for Open Science](https://osf.io/tvyxz/wiki/1.%20View%20the%20Badges/) badge for Preregistered. The materials are openly accessible at      . |
|  | This article has earned the [Center for Open Science](https://osf.io/tvyxz/wiki/1.%20View%20the%20Badges/) badge for Preregistered+. The materials are openly accessible at      . |
|  | This article has earned the [Center for Open Science](https://osf.io/tvyxz/wiki/1.%20View%20the%20Badges/) badges for Open Data and Open Materials through Open Practices Disclosure. The data and materials are openly accessible at https://doi.org/10.57760/sciencedb.35497        and      . |
|  | This article has earned the [Center for Open Science](https://osf.io/tvyxz/wiki/1.%20View%20the%20Badges/) badges for Open Data and Preregistered. The data and materials are openly accessible at       and      . |
|  | This article has earned the [Center for Open Science](https://osf.io/tvyxz/wiki/1.%20View%20the%20Badges/) badges for Open Data and Preregistered+. The data and materials are openly accessible at       and      . |
|  | This article has earned the [Center for Open Science](https://osf.io/tvyxz/wiki/1.%20View%20the%20Badges/) badges for Open Materials and Preregistered. The materials are openly accessible at       and      . |
|  | This article has earned the [Center for Open Science](https://osf.io/tvyxz/wiki/1.%20View%20the%20Badges/) badges for Open Materials and Preregistered+. The materials are openly accessible at       and      . |
|  | This article has earned the [Center for Open Science](https://osf.io/tvyxz/wiki/1.%20View%20the%20Badges/) badges for Open Data, Open Materials and Preregistered. The data and materials are openly accessible at      ,       and      . |
|  | This article has earned the [Center for Open Science](https://osf.io/tvyxz/wiki/1.%20View%20the%20Badges/) badges for Open Data, Open Materials and Preregistered+. The data and materials are openly accessible at      ,       and      . |
